# Association of Dietary Proportions of Macronutrients with Visceral Adiposity Index: Non-Substitution and Iso-Energetic Substitution Models in a Prospective Study

**DOI:** 10.3390/nu7105436

**Published:** 2015-10-26

**Authors:** Nazanin Moslehi, Behnaz Ehsani, Parvin Mirmiran, Parvane Hojjat, Fereidoun Azizi

**Affiliations:** 1 Nutrition and Endocrine Research Center, Research Institute for Endocrine Sciences, Shahid Beheshti University of Medical Sciences, Tehran 19395-4763, Iran; moslehinazanin@yahoo.com (N.M.); parvanehojjat@yahoo.com (P.H.); 2 Department of Clinical Nutrition and Dietetics, Faculty of Nutrition Sciences and Food Technology, National Nutrition and Food Technology Research Institute, Shahid Beheshti University of Medical Sciences, Tehran 19395-4741, Iran; behi.nutrist@gmail.com; 3 Endocrine Research Center, Research Institute for Endocrine Sciences, Shahid Beheshti University of Medical Sciences, Tehran 19395-4763, Iran; azizi@endocrine.ac.ir

**Keywords:** macronutrients, visceral adiposity index, visceral adiposity dysfunction, substitution model

## Abstract

We aimed to investigate associations between dietary macronutrient proportions and prospective visceral adiposity index changes (ΔVAI). The study included 1254 adults (18–74 years), from the Tehran Lipid and Glucose Study (TLGS), who were followed for three years. Dietary intakes were assessed twice using food frequency questionnaires. Associations of dietary macronutrient with ΔVAI and risk of visceral adiposity dysfunction (VAD) after three years were investigated. The percentage of energy intake from protein in the total population, and from fat in women, were associated with higher increases in VAI. A 5% higher energy intake from protein substituted for carbohydrate, monounsaturated fatty acids (MUFAs), and polyunsaturated fatty acids (PUFAs) was associated with higher ΔVAI. Higher energy intake from animal protein substituted for PUFAs was positively associated with ΔVAI. Substituting protein and PUFAs with MUFAs were related to higher ΔVAI. The associations were similar in men and women, but reached significance mostly among women. Risk of VAD was increased when 1% of energy from protein was replaced with MUFAs. Substituting protein for carbohydrate and fat, and fat for carbohydrate, resulted in increased risk of VAD in women. Higher dietary proportions of protein and animal-derived MUFA may be positively associated with ΔVAI and risk of VAD.

## 1. Introduction

Abdominal obesity is associated with increased risk of metabolic and cardiovascular diseases and mortality rates [1]. The effect of abdominal obesity on metabolic factors seems to be mainly mediated by the visceral adipose tissue (VAT) [2]. Both VAT and subcutaneous adipose tissue (SAT) have associated with metabolic risk factors, but only VAT has shown independent associations with metabolic risk factors after adjusting for body mass index (BMI) and waist circumference (WC) [3]. In addition to genetics, sex, and age, modifiable factors including physical activity and diet have been associated with VAT [4,5]. VAT and SAT seem to be differentially influenced by diet [5,6,7,8]. It has been suggested that diet can explain more of the variation in VAT compared to SAT [5], and lifestyle modification can reduce VAT more than it can reduce SAT [9]. Despite existing evidence suggesting VAT is predominately affected by the non-caloric qualitative aspects of diet, little is known about the association between macronutrient composition of diet and VAT [5,8].

Although magnetic resonance imaging (MRI) and computed tomography (CT) scans are considered as the gold standard for measuring VAT, their applicability to assess VAT is, however, limited because of their cost and availability. Therefore, different surrogates of visceral adiposity such as WC, BMI, and waist to hip ratio (WHR) are used to indirectly assess VAT in epidemiologic studies [10]. Visceral adiposity index (VAI) is a recently-proposed surrogate of visceral adiposity that can predict insulin resistance and cardio-metabolic risk factors [11,12,13]. VAI can be simply determined and is highly correlated with direct measurement of VAT [12,13].Considering both the anthropometric (WC and BMI) and the functional markers of visceral adiposity (triglycerides and HDL) to calculate VAI may improve the predictability of this score for occurrence of chronic disease [11]. To our knowledge, no observational study has to date examined the relationship of dietary intakes and VAI. This study aimed to investigate the association of dietary macronutrient composition with three-year VAI change (ΔVAI) and risk of visceral adiposity dysfunction (VAD) using both the non-substitution and substitution statistical approaches.

## 2. Methods

### 2.1. Study Population

Tehran Lipid and Glucose Study (TLGS) is an ongoing prospective study that was designed to investigate the risk factors for non-communicable disease among a representative sample of residents of district 13 of Tehran, the capital of Iran. The TLGS began in March 1999 and follow-up examinations have been conducted every three years to update information on demographic, lifestyle, medical, and biochemical variables. Detailed information on the TLGS study has been previously published [14]. The study was approved by the ethics committee of the Research Institute for Endocrine Sciences of Shahid Beheshti University of Medical Sciences, and written informed consent was obtained from each participant.

Of the 12523 participants in the third examination cycle (2006–2008), considered as the baseline for the current study, 4920 participants were randomly selected for dietary assessment, and 3462 participants completed the dietary assessment. For this study, 3150 participants aged 18–75 years were recruited. Pregnant and lactating women at either the third (baseline) or fourth examination cycles (follow-up; 2009–2011) (*n* = 128), individuals with chronic diseases (diabetes, cancer, and cardiovascular disease), and those taking drugs affecting anthropometrics or lipid profile variables (corticosteroid, lipid lowering drugs, and other hormonal drugs) (*n* = 687) were excluded. Individuals with missing data on dietary variables at follow-up (*n* = 561), and anthropometric or lipid profile variables at either baseline or follow-up were also excluded (*n* = 497). After further exclusion of those with extreme sex-specific energy intakes (>4200 kcal for women and >5000 kcal for men) equal to >2.5 SD (*n* = 23), 1254 participants remained for the analyses.

### 2.2. Dietary Assessment

Habitual dietary intakes over the previous year were assessed by a validated semi-quantitative 168-item food frequency questionnaire (FFQ) [15,16]. The frequency of consumption was given on a daily, weekly or monthly basis depending on the food items. Daily food intake (g/day) was estimated by multiplying the frequency of intake with portion sizes. Intakes of macronutrients (carbohydrate, fat, and protein), macronutrient subtypes (animal fat, vegetable fat, saturated fatty acids (SFAs), monounsaturated fatty acids (MUFAs), and polyunsaturated fatty acids (PUFAs), animal protein, and vegetable protein) were the exposures of the present study. To reduce within-person variation and also to represent the usual dietary intakes, a cumulative average intake of nutrients from FFQ at the third and the fourth examinations were used. In a random subset of the study participants, validity of the FFQ was evaluated against twelve 24-h dietary recalls and biomarkers [15].

### 2.3. Demographic, Lifestyle and Anthropometric Measurements

Data on demographic variables, smoking status (yes/no), past medical history, and drug use were obtained using a pre-tested questionnaire. Participants who smoked daily or occasionally were considered smokers and those who had never smoked, as non-smokers. Physical activity (PA) during the previous year was evaluated using the Modifiable Activity Questionnaire (MAQ), and was expressed as metabolic equivalent minute per week (MET-min/week) [17].

Weight and height were measured with participants wearing light clothing, without shoes, accurate to within 100 g and 0.5 cm. BMI was calculated as weight (kg) divided by height (m^2^). WC was measured to the nearest 0.1 cm at the midpoint between the bottom of the rib cage and top of iliac crest using a tape meter.

### 2.4. Biochemical Measurements

Blood was collected after 12–14 h overnight fasting. Triglycerides (TGs) were measured by enzymatic colorimetric analysis with glycerol phosphate oxidase. HDL-C was measured after precipitation of the apolipoprotein β containing lipoprotein with phosphotungistic acid. Analyses were performed using Pars Azmun kits (Pars Azmun Inc., Tehran, Iran) and a Selectra 2 autoanalyzer (Vital Scientific, Spankeren, The Netherlands). Inter- and intra-assay coefficients of variation (CVs) were 0.6% and 1.6% for TGs, and 0.5% and 2% for HDL-C.

### 2.5. Outcomes

The outcomes in the present study were three-year ΔVAI and the risk of VAD over three years. Since there was no considerable difference in follow-up time, absolute changes in VAI over the entire follow-up duration was considered as the outcome. VAI was calculated using sex-specific formulas: males [WC/39.68 + (1.88 × BMI)] × (TGs/1.03) × (1.31/HDL); Females: [WC/36.58 + (1.89 × BMI)] × (TGs/0.81) × (1.52/HDL), where both TGs and HDL levels are expressed in mmol/L [11]. The ΔVAI during three years of follow-up was determined by subtracting the baseline value from the follow-up value. Visceral adiposity dysfunction (VAD) was defined if the VAI score was greater than the age-specific optimal cut-off points of VAI [18].

### 2.6. Statistical Methods

Characteristics of the participants according to sex are summarized as mean ± SD for continuous variables or median (25th–75th percentiles) if continuous variables were skewed, and as proportions for categorical variables. Characteristics of women and men were compared, using *t*-test and Mann-Whitney for continuous variables, and chi-square for categorical variables. The intakes of macronutrients and macronutrient subtypes were adjusted for energy intake, using the nutrient density method, and expressed as per 5% increase in energy intake. Multivariate linear regression analyses were used to investigate the single effect of increase in each nutrient intake as a continuous variable and the subsequent three-year ΔVAI. In these analyses the following demographic and lifestyle covariates were included in model one: baseline age (continuous), sex (except sex strata), physical activity (continuous), smoking (yes/no), baseline VAI (continuous). To evaluate the independent effect of each nutrient when total energy and other nutrients were constant the following dietary intakes data were further included in model two: intakes of total energy, cholesterol, and other contributing macronutrients (continuous). For analyses on subtypes of protein and fat (animal and vegetable sources), two subtypes of protein and fat were mutually adjusted by including two subtypes simultaneously in model two. To determine the independent associations of SFAs, MUFAs, and PUFAs, the multivariate linear regression model two included SFAs, MUFAs, and PUFAs simultaneously instead of total fat and then adjusted for the same covariates as in the model for total fat. Effect modification by sex was investigated by including a cross-product interaction term between the exposure variables (continuous) and sex in the fully adjusted model (model two).

Substitution models were performed to estimate the effects of iso-caloric substituting 5% of energy from one type of macronutrient by 5% of energy from another macronutrient, using multivariate nutrient density models. The coefficients in these models can be interpreted as estimated change in VAI by 5% increase in one nutrient at the expense of another not included in the model, while keeping total energy intake and other nutrients, which are included in the model, constant.

Multivariate-adjusted logistic regression analyses were used to estimate the odds of occurrence of VAD after replacement of one macronutrient with another macronutrient (substitution model) in individuals without VAD at baseline (*n* = 795). All substitution models were adjusted for the above-mentioned demographic and lifestyle covariates, and intakes of total energy and cholesterol. For these analyses odds ratios (95% CIs) were estimated for replacement of 1% of energy from one nutrient with another nutrients. All statistical analyses were performed with SPSS (Version 15.0; Chicago, IL, USA) and *p*-values < 0.05 were considered significant.

## 3. Results

Characteristics and dietary intakes of the participants, according to the sex, are presented in Table 1. Compared to women, men were more active, more likely to be smokers, and had higher WC, VAI, and TGs, but lower HDL-C at baseline and follow-up examinations. ΔVAIs after three years follow-up were not significantly different between two sexes. The average percentage of energy from carbohydrate was higher in men, while the percentage of fat intake was higher in women. While there was no significant difference in the percentage of protein intakes between men and women, intakes of animal protein were significantly higher in women than men. The percentage of energy from SFA, MUFA and PUFA were also significantly higher in women than men.

**Table 1:** Characteristics and dietary intakes of the participants.

Variables	Women (*n* = 635)	Men (*n* = 619)	*p*-Value
Baseline age (year)	36 (26, 45)	37 (27, 47)	0.04
Physical activity (Met-min/week)	184.6 (46.5, 632.2)	199. 5 (83.4, 952.6)	<0.001
Smoker (%)	20 (3.1%)	153 (24.7%)	<0.001
Body mass index (kg/m^2^)			
Baseline	26.3 ± 5.3	26.4 ± 4.3	0.83
Follow-up	27.3 ± 5.4	26.9± 4.2	0.17
Waist circumference (cm)			
Baseline	82. 5 ± 13.1	93.4 ± 11.2	<0.001
Follow-up	88.8 ± 12.5	95.7 ± 11.0	<0.001
Visceral adiposity index			
Baseline	1.68 (1.12, 2.60)	1.92 (1.23,3.05)	0.001
Follow-up	1.55 (1.04, 2.47)	1.82 (1.17,2.76)	<0.001
3-Year change	−0.19 ± 1.21	−0.26 ± 1.38	0.30
Triglycerides (mmol/L)			
Baseline	1.10 (0.81, 1.60)	1.45 (0.99, 2.01)	<0.001
Follow-up	1.06 (0.80, 1.54)	1.47 (1.00, 2.00)	<0.001
HDL (mmol/L)			
Baseline	1.18 ± 0.26	1.00 ± 0.21	<0.001
Follow-up	1.35 ± 0.30	1.10 ± 0.23	<0.001
Daily dietary intakes			
Energy (Kcal)	2267 ± 654	2629 ± 761	<0.001
Carbohydrates (g)	323 ± 100	389± 117	<0.001
Fat (g)	80.6 ± 29.9	85.8 ± 30.4	0.002
Protein (g)	80.7 ± 27.0	93.1 ± 29.6	<0.001
Carbohydrates (% of energy)	57.1 ± 6.0	59.3 ± 5.3	0.001
Fat (% of energy)	31.9 ± 5.9	29.2 ± 4.9	<0.001
Protein (% of energy)	14.3 ± 2.6	14.2 ± 1.9	0.40
Animal fat (% of energy)	14.3 ± 4.3	13.9 ±4.3	0.060
Plant fat (% of energy)	17.6 ± 6.1	15.5 ± 4.1	<0.001
Animal protein (% of energy)	7.44 ± 2.55	7.11 ± 2.21	0.019
Plant protein (% of energy)	6.17 ± 1.25	6.60 ± 1.22	<0.001
SFAs (% of energy)	10.4 ± 2.4	9.9 ± 4.7	0.01
PUFAs (% of energy)	6.5 ± 1.9	5.9 ± 1.5	<0.001
MUFAs (% of energy)	10.8 ± 2.6	9.8 ± 1.9	<0.001

Values are mean ± SD, median (25th, 75th percentiles), or *n* (percentage). SFAs, saturated fatty acids; MUFAs, monounsaturated fatty acid; PUFAs, polyunsaturated fatty acids.

Estimated effects of nutrient intakes on three-year ΔVAI are presented in Table 2. In women, a 5% increase in intake of fat was positively associated with changes in VAI after adjusting for other energy contributing macronutrients (model 2; β: 0.187; *p* = 0.048). The percentage of protein intake was positively associated with ΔVAI in men and women combined; each 5% higher percentage of protein intakes was associated with a 0.203 higher increase in VAI after adjustment for all relevant confounders. (*p* = 0.018). In separate analyses, based on the sex, this association was significant among women only (β: 0.266; *p* = 0.010).

**Table 2:** Estimated effects of nutrient intakes on 3-year visceral adiposity index change.

Type of Nutrient	All (*n* = 1254)		Women (*n* = 635)		Men (*n* = 619)	
	β	*p*-value	β	*p*-value	β	*p*-value
Carbohydrate (5% of energy)						
Model 1 ^1^	−0.003	0.904	−0.013	0.700	0.012	0.782
Model 2 ^2^	0.097	0.199	0.140	0.130	−0.017	0.901
Fat (5% of energy)						
Model 1	0.008	0.765	0.028	0.432	−0.025	0.591
Model 2	0.118	0.136	0.187	0.048	−0.036	0.803
Protein (5% of energy)						
Model 1	0.099	0.129	0.109	0.168	0.086	0.454
Model 2	0.203	0.018	0.266	0.010	0.091	0.572
Animal fat (5% of energy)						
Model 1	0.014	0.696	0.018	0.712	0.006	0.916
Model 2 ^3^	0.084	0.275	0.090	0.338	0.062	0.665
Plant fat (5% of energy)						
Model 1	0.005	0.859	0.017	0.616	−0.021	0.693
Model 2 ^4^	0.083	0.536	0.091	0.203	0.046	0.736
SFAs (5% of energy)						
Model 1	−0.006	0.879	0.068	0.433	−0.032	0.500
Model 2 ^5^	−0.011	0.820	0.090	0.498	−0.026	0.620
MUFAs (5% of energy)						
Model 1	0.106	0.107	0.141	0.072	0.023	0.849
Model 2 ^6^	0.229	0.067	0.231	0.138	0.153	0.562
PUFAs (5% of energy)						
Model 1	0.082	0.347	0.089	0.407	0.058	0.696
Model 2 ^7^	0.019	0.904	−0.011	0.962	0.092	0.711
Animal protein (5% of energy)						
Model 1	0.043	0.498	0.002	0.922	0.096	0.333
Model 2 ^8^	0.178	0.123	0.173	0.239	0.227	0.241
Plant protein (5% of energy)						
Model 1	−0.040	0.746	−0.036	0.827	−0.037	0.840
Model 2 ^9^	0.051	0.757	0.079	0.712	0.025	0.925

^1^ Model 1 adjusted for age (continuous), sex (except sex strata), smoking (yes/no), physical activity (continuous), and baseline VAI (continuous). ^2^ Adjusted for intakes of total energy (continuous), cholesterol (continuous), and the percentage of the other energy contributing macronutrients (all continuous). ^3^ Additionally adjusted for plant fat (% of energy) instead of total fat. ^4^ Additionally adjusted for the animal fat (% of energy) instead of total fat. ^5^ Additionally adjusted for MUFAs and PUFAs (% of energy) instead of total fat. ^6^ Additionally adjusted for SFAs and PUFAs (% of energy) instead of total fat. ^7^ Additionally adjusted for SFAs and MUFAs (% of energy) instead of total fat. ^8^ Additionally adjusted for plant protein (% of energy) instead of total protein. ^9^ Additionally adjusted for animal protein (% of energy) instead of total protein. SFAs, saturated fatty acids; MUFAs, monounsaturated fatty acid; PUFAs, polyunsaturated fatty acids.

Adjusted three-year ΔVAI for the iso-energetic increase of 5% of energy from one macronutrient at the expense of another macronutrient are presented in Table 3. A 5% higher energy intake from protein substituted for carbohydrates, MUFAs, and PUFAs was significantly associated with higher increases in VAI in men and women combined. These associations were similar for men and women, but reached statistical significance only among women. A 5% higher proportion of animal protein replaced PUFA was associated with 0.166 higher increases in VAI in men and women combined, but the association was significant only in men. The substitution of protein and PUFAs by MUFAs was positively associated with ΔVAI in men and women combined, the associations being significant only in women. No other substitutions were significantly associated with VAI change.

**Table 3:** Adjusted 3-year visceral adiposity index (VAI) change for the iso-energetic increase (↑) of 5% of energy from one macronutrient at the expense of another (↓) ^1^.

Nutrient Substitution	All (*n* = 1254)		Women (*n* = 635)		Men (*n* = 619)	
	β	*p*-value	β	*p*-value	β	*p*-value
Carbohydrate ↑						
Fat ↓^2^	−0.007	0.807	−0.027	0.478	0.015	0.746
Protein ↓^3^	0.003	0.963	0.041	0.630	−0.066	0.521
Fat ↑						
Carbohydrate ↓^2^	0.024	0.428	0.056	0.146	−0.019	0.697
Protein ↓^4^	0.010	0.882	−0.088	0.419	0.060	0.458
Protein ↑						
Carbohydrate ↓ ^3^	0.145	0.048	0.201	0.033	0.104	0.398
Fat ↓ ^4^	0.129	0.067	0.160	0.071	0.117	0.340
MUFAs ↓ ^4,5,6^	0.174	0.021	0.218	0.021	0.159	0.232
PUFAs ↓ ^4, 5,7^	0.155	0.029	0.183	0.039	0.157	0.229
Animal protein ↑						
Carbohydrate ↓ ^3,8^	0.079	0.322	0.055	0.518	0.154	0.224
Fat ↓ ^4,8^	0.077	0.328	0.028	0.779	0.193	0.145
Plant protein ↓ ^3,4^	0.163	0.117	0.152	0.261	0.218	0.187
MUFAs ↓ ^4,5,6,8^	0.147	0.102	0.098	0.397	0.289	0.055
PUFAs ↓ ^4,5,7,8^	0.166	0.050	0.122	0.257	0.299	0.048
Plant protein ↑						
Carbohydrate ↓ ^3,9^	0.004	0.981	0.040	0.851	−0.021	0.931
Fat ↓ ^4,9^	−0.027	0.858	−0.018	0.929	−0.006	0.980
Animal protein ↓ ^3,4^	−0.062	0.678	−0.019	0.923	−0.137	0.542
MUFA ↓ ^4,5,6,9^	−0.024	0.878	0.024	0.908	−0.010	0.967
PUFA ↓ ^4,5,7,9^	0.002	0.992	0.019	0.927	0.050	0.835
Animal fat ↑						
Carbohydrate ↓ ^2,10^	0.018	0.693	0.039	0.542	0.000	1.00
Protein ↓ ^4,10^	0.070	0.364	0.088	0.349	0.024	0.863
Plant fat ↓ ^2,4^	0.008	0.874	0.008	0.910	0.022	0.782
Plant fat ↑						
Carbohydrate ↓ ^2,11^	0.025	0.432	0.046	0.231	−0.014	0.813
Protein ↓ ^4,11^	0.035	0.555	0.047	0.487	−0.015	0.899
MUFA ↑						
Carbohydrate ↓ ^2,5,6^	0.171	0.149	0.232	0.136	0.008	0.534
Protein ↓ ^4,5,6^	0.260	0.037	0.306	0.043	0.113	0.665
PUFAs ↓ ^2,4,5^	0.239	0.013	0.226	0.038	0.213	0.304
PUFA ↑						
Carbohydrate ↓ ^2,5,7^	−0.041	0.785	−0.112	0.569	0.088	0.724
Protein ↓ ^4,5,7^	−0.096	0.516	−0.178	0.381	0.024	0.922
MUFAs ↓ ^2,4,5^	0.204	0.090	0.226	0.145	0.181	0.355

^1^ All models adjusted for age (continuous), sex (except sex strata), smoking (yes/no), physical activity (continuous), and baseline VAI (continuous), total energy intake (continuous), and cholesterol intake (continuous). ^2^ additionally adjusted for protein (% of energy). ^3^ additionally adjusted for fat (% of energy). ^4^ additionally adjusted for carbohydrate (% of energy). ^5^ additionally adjusted for SFA. ^6^ additionally adjusted for PUFA (% of energy). ^7^ additionally adjusted for MUFA (% of energy). ^8^ additionally adjusted for plant protein (% of energy). ^9^ additionally adjusted for animal protein. ^10^ additionally adjusted for plant fat (% of energy). ^11^ additionally adjusted for animal fat (% of energy). SFAs, saturated fatty acids; MUFAs, monounsaturated fatty acid; PUFAs, polyunsaturated fatty acids.

After excluding individuals with VAD at baseline, 82 participants (48 men and 34 women) showed VAD after a three-year follow-up. Adjusted odds ratios (95% CI) of VAD after three years according to the iso-energetic increase of 1% of energy from one nutrient at the expense of another nutrient are presented in Table 4. In women and men combined analyses, when 1% of energy from protein was replaced with the same amount MUFA, the odds of having VAD was increased by 21% (*p* = 0.038); although the association was similar for both genders, it was not significant in either. Replacing 1% of energy from carbohydrate with the same amount of fat (OR = 1.09; *p* = 0.008) and protein (OR = 1.24; *p* = 0.020) were significantly associated with increased risk of VAD in women. When 1% of energy from fat was replaced with protein, the risk of VAD increased by 20% in women. Replacing 1% of energy from fat, protein, and animal protein with 1% of energy from carbohydrate lowered the risk of VAD in women. None of the substitution models showed statistically significant associations with VAD in men. No significant interaction based on gender was observed.

**Table 4:** Odds ratios (OR) of visceral adiposity dysfunction after 3 years according to the iso-energetic increase of 1% of energy from one macronutrient at the expense of another.

Nutrient Substitution	All (*n* = 795)			Women (*n* = 420)			Men (*n* = 375)		
	OR	95% CI	*p*-value	OR	95% CI	*p*-value	OR	95% CI	*p*-value
Replacement of carbohydrate									
With fat	1.02	0.98, 1.07	0.326	1.09	1.02, 1.15	0.008	0.97	0.91, 1.04	0.349
With protein	1.08	0.95, 1.22	0.253	1.24	1.03, 1.48	0.020	1.05	0.88, 1.26	0.568
Replacement of fat									
With carbohydrate	0.97	0.93, 1.02	0.262	0.91	0.85, 0.97	0.004	1.03	0.96, 1.09	0.444
With protein	1.06	0.94, 1.19	0.349	1.20	1.01, 1.43	0.041	1.07	0.90, 1.27	0.438
Replacement of protein									
With carbohydrate	0.94	0.83, 1.05	0.259	0.81	0.67, 0.99	0.039	0.95	0.81, 1.12	0.532
With total fat	0.96	0.86, 1.07	0.446	0.88	0.74, 1.06	0.172	0.92	0.78, 1.09	0.335
With MUFAs	1.21	1.01, 1.45	0.038	1.27	0.99, 1.64	0.065	1.10	0.74, 1.63	0.640
With PUFAs	0.83	0.65, 1.06	0.135	0.79	0.57, 1.09	0.149	0.86	0.56, 1.32	0.490
Replacement of plant protein									
With carbohydrate	0.95	0.80, 1.13	0.561	0.83	0.62, 1.11	0.207	1.02	0.82, 1.27	0.887
With total fat	0.97	0.83, 1.14	0.726	0.90	0.67, 1.18	0.438	0.98	0.79, 1.20	0.811
With animal protein	1.02	0.85, 1.23	0.814	1.03	0.77, 1.36	0.854	1.11	0.87, 1.41	0.420
Replacement of animal protein									
With carbohydrate	0.93	0.84, 1.06	0.269	0.82	0.68, 0.99	0.039	0.96	0.83, 1.13	0.547
With total fat	0.96	0.85, 1.07	0.428	0.88	0.74, 1.06	0.175	0.91	0.77, 1.08	0.286
With plant protein	0.97	0.75, 1.23	0.835	1.02	0.713, 1.47	0.898	0.90	0.64, 1.27	0.550
Replacement of PUFAs									
With MUFAs	1.12	0.98, 1.27	0.103	1.12	0.95, 1.32	0.193	1.03	0.78, 1.37	0.836

All models adjusted for age (continuous), sex (except sex strata), smoking (yes/no), physical activity (continuous), and baseline VAI (continuous), total energy intake (continuous), and cholesterol intake(continuous). SFAs, saturated fatty acids; MUFAs, monounsaturated fatty acid; PUFAs, polyunsaturated fatty acids.

## 4. Discussion

In the present study, after adjusting for all potential confounders and other dietary intake variables, higher intake of protein was associated with higher increase in VAI after three years. In iso-energetic diet, replacing carbohydrate, MUFAs, and PUFAs with protein was positively associated with three-year ΔVAI. A 5% higher proportion of animal protein substituted for PUFA was also positively associated with ΔVAI. A 5% higher proportion of MUFA replaced either protein or PUFA was associated with higher ΔVAI. In sex stratified analyses, the direction of theses associations were similar in both men and women, but the associations were statistically significant, mostly among women. The risk of occurrence of VAD increased with increasing the proportion of MUFA intake substituted for protein intake in the total population, men and women combined. Substituting protein for carbohydrate and fat, and fat for carbohydrate resulted in significant increase in the risk of VAD in women. Since analyses were adjusted for total energy intake, our findings show the effect of changes in proportions of macronutrient in iso-energetic diet.

Among the limited observational studies available on the association between the dietary intake and visceral adiposity measured directly by MRI or CT scan, only two studies have so far investigated the association prospectively [7,19]. No significant association was reported between two-year changes in total protein intake and change in VAT in 85 overweight youths, aged 11–17 years [19]. Total protein intake was also not related to five-year percent change in VAT in 1114 black and Hispanic overweight adults in another prospective study [7]. Among cross-sectional studies of directly measures of VAT by MRI, one study showed a positive association with total protein intake [5] while other did not report any significant association [20,21,22]. None of these studies however evaluated the effect of substituting a proportion of any macronutrient intake with that of another. In line with our finding, observational studies have shown a positive association of protein intake with percent body fat [23,24,25]. In a prospective study, a higher intake of dietary protein, substituted for either carbohydrate or fat, was found to be related to increased body weight and BMI after a six-year follow-up, an increase mainly attributed to increased body fat mass [25]. Previous observational studies evaluating associations of protein intake with other surrogates of visceral adiposity including WC, WHR, and BMI reported positive, negative or even no associations [24,26,27,28,29,30]. Differences in the study size, study design, surrogates of visceral adiposity used, methods of collecting weight and WC data (self-reporting/measuring), statistical methods and analyses could be partly explain these heterogeneous findings. Satiating property and increased energy expenditure, which can promote weight loss, have been proposed for high protein diets in interventional studies. However, observational studies suggest that within the range of habitual intake in diets, protein has no long term satiating effect. The mechanism that dietary protein can affect adiposity is unknown but protein through its effects on insulin action may influence adiposity [25,31].

In our study, after adjusting for life style and all dietary intake variables (model two) fat intake was positively associated with the subsequent ΔVAI, only among women. An increase in the proportion of fat intake at the expense of other macronutrients in an iso-energetic diet showed no significant association with ΔVAI, while replacing carbohydrate with fat increased the risk of VAD over three year follow-up in women. In subgroup analyses, according to the source of fat, no significant associations of plant or animal fat with VAI were observed. Increasing MUFA by decreasing total protein or PUFA in iso-energetic diets was positively associated with ΔVAI. In previous observational studies evaluating the relation of fat intake and VAT [7,19,20,21,22], only one cross-sectional study reported a positive association between fat intake and VAT in overweight young adult aged 17–35 years [22]. Fat intake in the range of 18%–40% of energy intakes were showed to have little effect on body fatness [32]. The other aspects of fat intake including source of fat (plant *vs.* animal) and subtype of fatty acid have hardly been investigated. In a prospective study, no association was observed between SFAs, MUFAs, PUFAs, and five-year percent change of VAT [7]. Noteworthy, consumption of olives and olive oil in our population are very low and MUFA intakes in our study were mainly derived from animal sources. The positive association of MUFAs with VAI change was observed when MUFAs substituted for either protein or PUFAs intakes, independent of SFAs and cholesterol intakes. However, the confounding effect of other dietary components in these sources could not be ruled out. The hypothesis that MUFAs are healthy fatty acids comes from studies investigating the effects of olive oil, whereas further studies suggest MUFA intakes from animal sources to have different effects [33,34]. On the other hand, the health benefits of olive and olive oil may be attributed to other components rather than the contents of MUFAs [34]. There is some evidence suggesting that different isomeric profiles of MUFAs may have different metabolic consequences. The association observed in this study may be partly attributed to trans-MUFAs, which have been associated with reduced HDL, increased postprandial insulinemia and low-grade inflammation [33,35,36].

We did not observe any significant association between carbohydrate intake and ΔVAI in the non-substitution model. However, replacing carbohydrate with total protein was positively associated with ΔVAI, statistically significant in women only. While holding energy intake constant, the risk of VAD was reduced by 9% and 19%, respectively per each percentage increase in proportion of carbohydrate at the expense of fat and protein among women. Previous observational studies using a non-substitution approach did not find any significant association between carbohydrate intake and VAT [19,20,21,22]. However, consistent with our findings, prior observational studies using substitution models showed reduced risk of abdominal obesity by increases in carbohydrate intake at the expense of either fat or protein intake [37], and reduced weight gain with increasing carbohydrate at the expense of protein intake [31]. In an interventional study, PCOS women lost more total body fat following consumption of a reduced carbohydrate diet for eight weeks (41%:19%:40% energy from carbohydrate:protein:fat) compared to a standard diet (55%:18%:27%) [38]. In that study, the short-term effects of the diet have been examined, whereas we have investigated the habitual dietary intake of macronutrients in the long term. In addition, the effects of diet on PCOS women may differ in women without the condition.

Gender has been shown to be the most important determinant of visceral adiposity [5]. Due to the significant differences in visceral adiposity and dietary intakes in the both sexes, we repeated our analyses for men and women separately. Most of the significant associations observed in pooled men and women analyses, reached significance only among women. However, the directions of these associations were mostly similar for men and women and we found no significant interaction for sex. More precisely report of food intakes and higher variations in proportions of macronutrients in diet of women, compared with men partly explain the significant associations in women only. However, our findings may also indicate that the proportions of macronutrients in habitual diets are predictors of visceral adiposity and its function that are more important in women. A cross-sectional study suggested sex modifications in the associations between nutrients intakes and VAT measured by MRI [5]. More studies are needed to clarify the possible effect of gender on the association between macronutrients intakes and visceral adiposity and its function.

The prospective design of the study, using an FFQ specially validated in the population studied, and our repeated assessments of dietary intakes to control the possible changes of dietary intakes during follow-up are among strengths of the present study. Analyzing data using two different statistical approaches of non-substitution and substitution models add to the strengths mentioned. In non-substitution model, the association of the proportion of single nutrient in diet was investigated, independent of intakes of energy and other macronutrient intakes. In the substitution model, the effect of substituting one nutrient intake for another in diet was investigated while the energy intake keeps constant. Some limitations should also be mentioned; first, despite using a validated FFQ in this study, FFQs, like other tools of self-reporting assessments of dietary intakes, are subject to measurement errors. Second, in this study, the associations of proportions of macronutrients in habitual diets with visceral adiposity were investigated indirectly using VAI as a surrogate of visceral adiposity. There are no definite cut-off points of VAI to diagnose visceral adiposity in all populations. In this study, the cut-off points used to identify VAD were based on the age-stratified cutoff points proposed among Caucasian populations, which were strongly associated with cardio-metabolic risk factors [18]. However, one study conducted on our population also confirmed that the best cutoff point of VAI associated with cardiovascular disease is around of 2.2 very similar to that of Caucasian populations [39]. Third, the study participants are not representative of the whole Iranian population, which limits the generalizability of our findings to other Iranian populations and to some other non-Iranian populations that have different proportions of macronutrients in their habitual diet compared to our study participants.

## 5. Conclusions

In conclusion, our findings suggest that the proportions of macronutrient intake in habitual diets may be associated with visceral adiposity and its function, defined as VAI, independent of total energy intake. Higher proportions of total protein and animal-derived MUFA in diet may be positively associated with higher increase in VAI and risk of VAD during three years of follow-up, especially among women.
